# Supplementary material for: Cre/lox-assisted non-invasive in vivo tracking of specific cell populations by positron emission tomography
Source: Nat Commun. 2017 Sep 5;8:444. doi: 10.1038/s41467-017-00482-y (PMC5585248; doi:10.1038/s41467-017-00482-y)
Supplement: Supplementary file 1 — Supplementary Information [file 41467_2017_482_MOESM1_ESM.pdf]

File name: Supplementary Information
Description: Supplementary figures, supplementary table and supplementary references.

File name: Peer review file
Description:

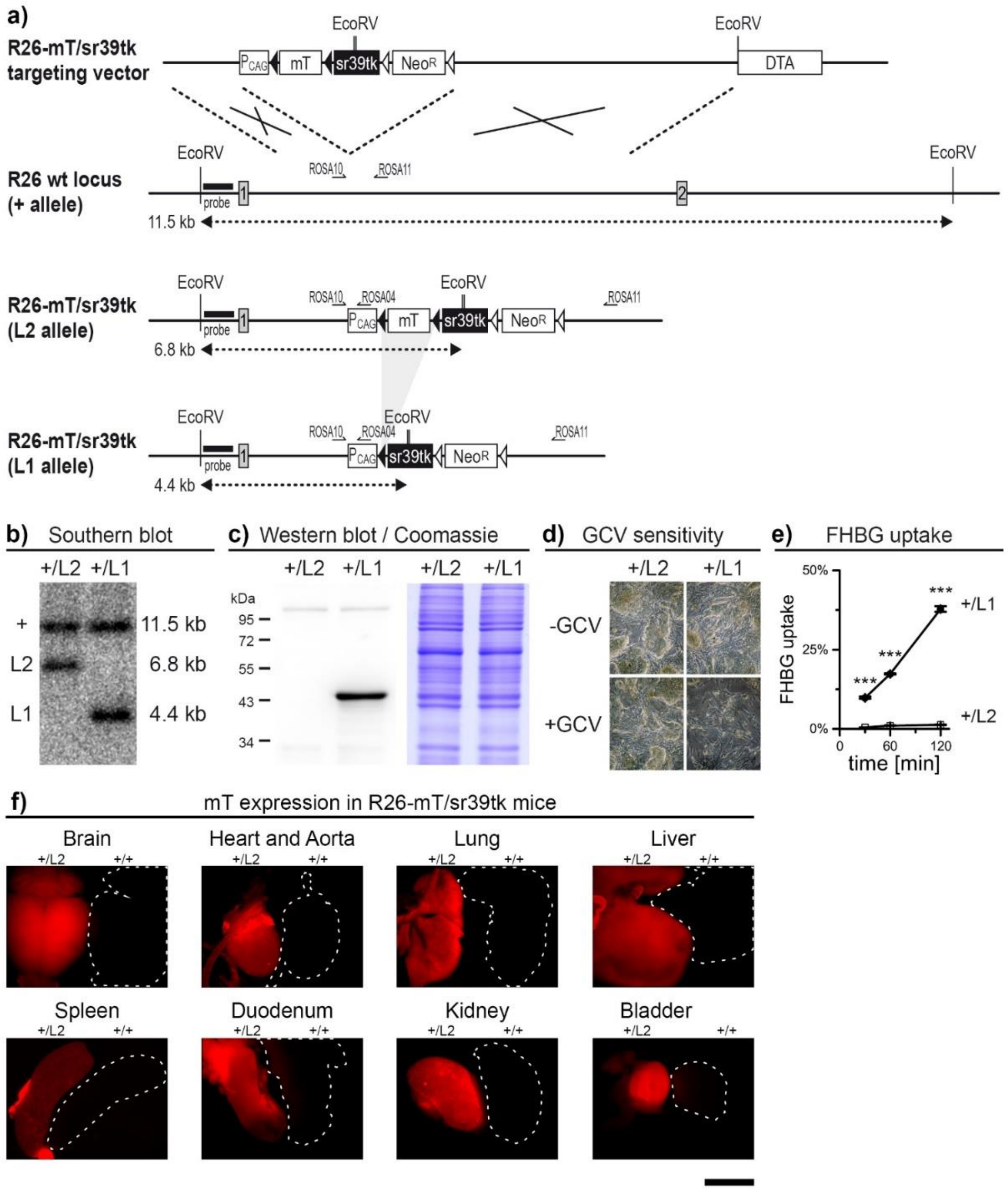

Supplementary Figure 1. Generation of R26-mT/sr39tk PET reporter mice. (**a**) Gene targeting strategy to insert a Cre-activatable sr39tk construct into the intron between exon 1 and 2 (grey boxes) of the murine ROSA26 locus (wild type allele indicated by '+'). The CAG promoter (P_{CAG}) drives expression of membrane-targeted tandem dimer tomato red fluorescent protein (mT) before Cre recombination (L2 allele with two loxP sites; black arrowheads indicate loxP sites) and expression of sr39tk after Cre recombination (L1 allele with one remaining loxP site). A FRT-flanked (white arrowheads) neomycin resistance cassette (Neo^R) and a diphtheria toxin cassette (DTA) were used for selection of ES cells. For Southern blot analysis, EcoRV was used together with a probe located 5‘ to the integration site resulting in fragment lengths of 11.5 kb for the wild type (+) allele, 6.8 kb for the L2 allele, and 4.4 kb for the L1 allele. Primers ROSA10, ROSA11, and ROSA04 were used for PCR genotyping of R26-mT/sr39tk mice. (**b-e**) Characterization of R26-mT/sr39tk ES cells. (**b**) Southern Blot of EcoRV-digested genomic DNA of a targeted ES cell clone before (+/L2 genotype, left lane) and after Cre-mediated excision of the loxP-flanked mT

cassette (+/L1 genotype, right lane). The expected positions of the DNA fragments derived from the respective alleles are indicated to the right. (**c**) Analysis of sr39tk protein expression before (+/L2 genotype, left lane) and after (+/L1 genotype, right lane) Cre-mediated recombination. Protein lysates (20 µg) from ES cells with +/L2 or +/L1 genotype were subjected to SDS-PAGE followed by Western blot analysis (left) with a rabbit polyclonal HSV1-tk antiserum (1:2000 dilution, provided by W.C. Summers). The positions of protein weight markers are indicated to the left. Coomassie staining of the gel (right) was used to confirm equal loading. (**d**) As a first test of the functionality of the sr39tk protein expressed from the L1 allele, a ganciclovir (GCV) sensitivity assay was performed. GCV is a nucleoside analogue that is activated by *HSV1*-tk-mediated phosphorylation and then leads to inhibition of DNA replication and cell death. ES cells with +/L2 or +/L1 genotype were grown in normal medium (-GCV) or medium containing 2 µM GCV (+GCV). Images were acquired 5 days after beginning of the treatment and show almost complete ablation of GCV-treated +/L1 ES cells, while growth of +/L2 cells was not affected by GCV. (**e**) As a second test of functionality, a [^{18}F]FHBG uptake assay was performed. ES cells with +/L2 or +/L1 genotype were incubated in medium containing 740 kBq/mL [^{18}F]FHBG. Uptake of radioactivity into the cells was determined at 30, 60, and 120 min. FHBG uptake is given as ratio between intracellular and total activity in the sample (mean±s.e.m. of three 6-wells for every time point). Stars indicate significant differences between genotypes (***, $p<0.001$, 1-way ANOVA). (**f**) Expression of mT in R26-mT/sr39tk mice; mT-derived fluorescence of organs isolated from R26-mT/sr39tk (+/L2; genotype: Cre[+/+],R26[sr39tk/+]) was recorded using a fluorescence stereoscope. To control for background fluorescence, organs isolated from wild-type mice (+/+; genotype: Cre[+/+],R26[+/+]; white outlines) were placed next to the corresponding organ of the R26-mT/sr39tk mice. Scale bar: 1 cm.

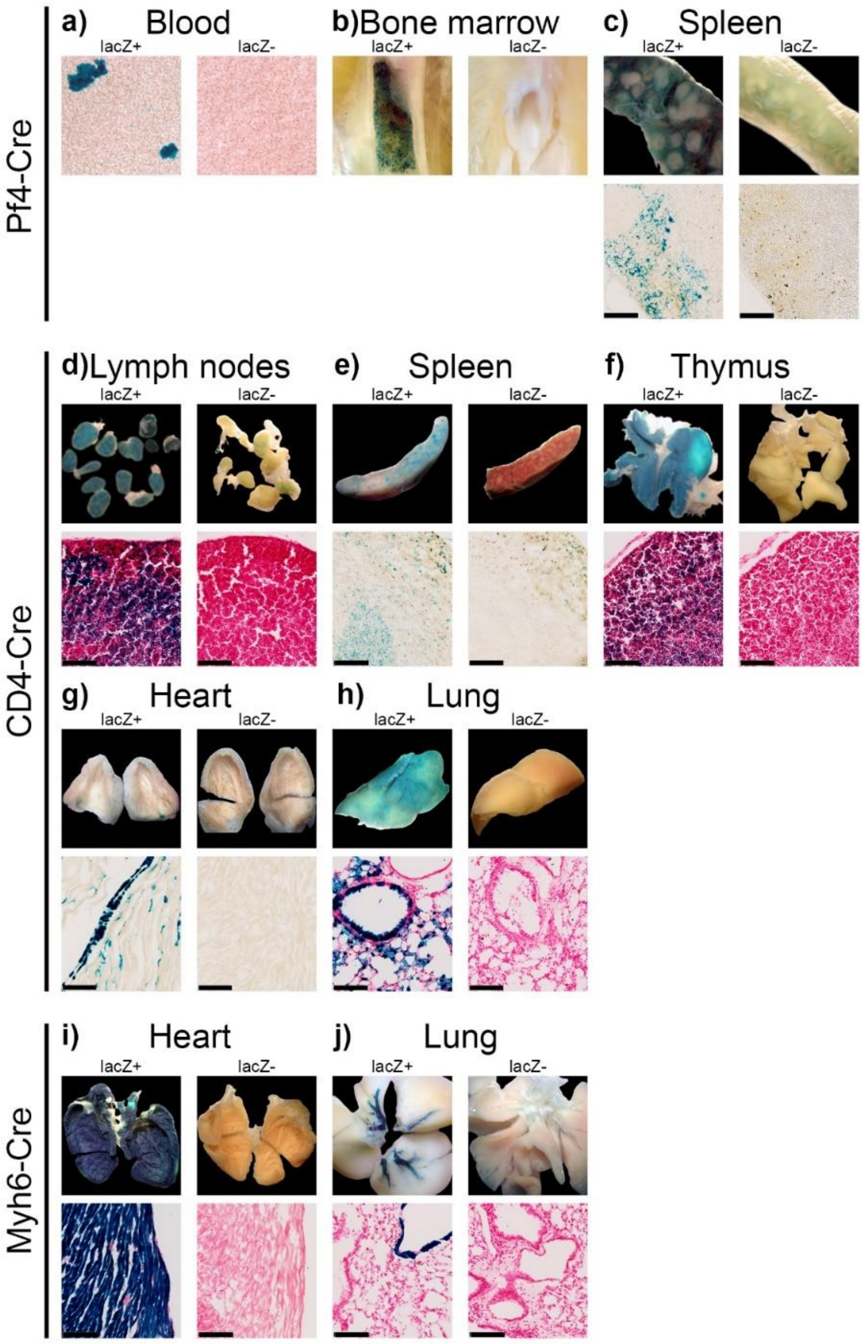

Supplementary Figure 2. Analysis of Cre recombinase activity with R26-lacZ Cre reporter mice. Tissues were from (**a-c**) Pf4/lacZ mice, (**d-h**) CD4/lacZ mice, and (**i, j**) Myh6/lacZ mice. Panels show X-Gal-stained whole-mounts of organs (upper rows) and 20-µm tissue sections (lower rows) of the respective Cre/lacZ mice (lacZ+; genotype: Cre[tg/+],R26[lacZ/+]) and litter-matched Cre-negative control animals (lacZ-; genotype: Cre[+/+],R26[lacZ/+]); panel **a** shows microscopic images of X-Gal-stained whole blood. X-Gal-positive cells are blue. Sections in **d, f, h, i, j** were counterstained with nuclear fast red; sections in **c, e, g** were not counterstained. Similar results were obtained with organs from ≥3 animals of each genotype. Scale bars: 100 µm.

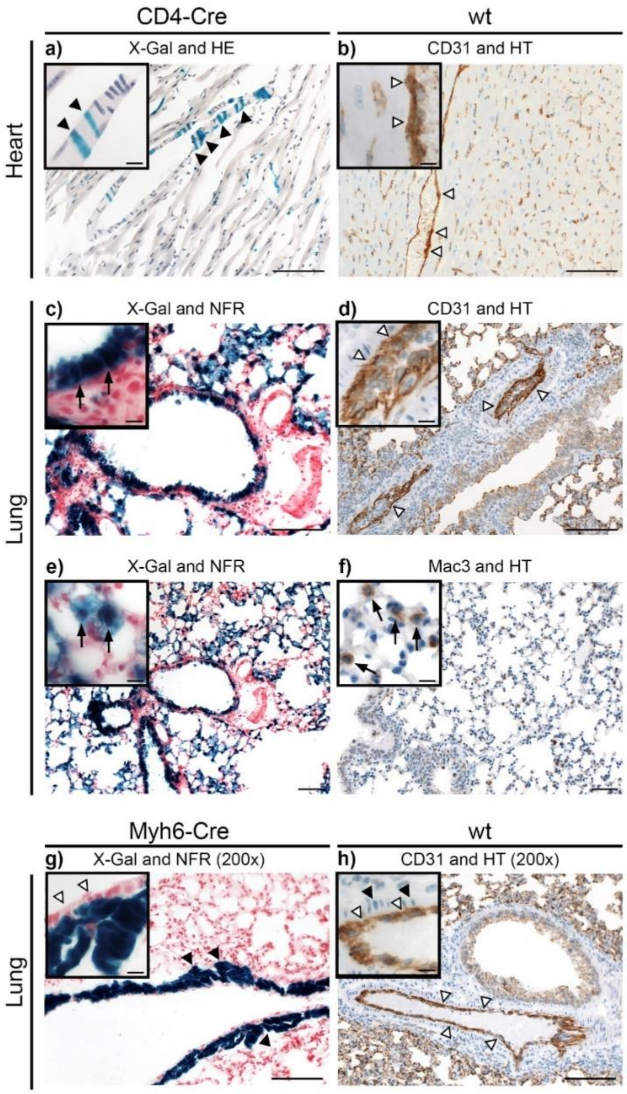

Supplementary Figure 3. Detailed analysis of ectopic recombination in CD4-Cre and Myh6-Cre mouse lines. X-Gal-stained sections of the heart and lung from (**a, c, e**) CD4/lacZ mice and (**g**) Myh6/lacZ mice. Sections were counterstained with hematoxylin/eosin (HE) or nuclear fast red (NFR). X-Gal-positive cells are blue. For X-Gal staining of Cre-negative controls, see Supplementary Fig. 3. To determine the identity of X-Gal-stained cells, sections of (**b**) heart and (**d, f, h**) lung from wild type (wt) mice were immunostained with antibodies against the endothelial cell marker CD31 or the macrophage marker Mac3 as indicated. These sections were counterstained with hematoxylin (HT). Antibody-stained cells are brown. In panel **a**, closed arrowheads point to X-Gal-positive smooth muscle cells of medium-sized vessels of the myocardium. In panel **c** and **e**, X-Gal-positive cells indicated by arrows represent bronchial epithelial cells and alveolar macrophages, respectively. In panel **f**, alveolar macrophages are stained with the macrophage marker Mac3 (arrows). In panel **g**, X-Gal-positive cells are smooth muscle cells of the lung vasculature (closed arrowheads). Note that endothelial cells (inset, open arrowheads) are negative for X-Gal. In contrast, endothelial cells, which are indicated by open arrowheads in panel **b**, **d** and **h**, are positive for the endothelial-specific marker CD31, whereas smooth-muscle cells (closed arrowheads in **h**) are not stained for CD31. Scale bars: panels, 100 µm; insets, 1 µm.

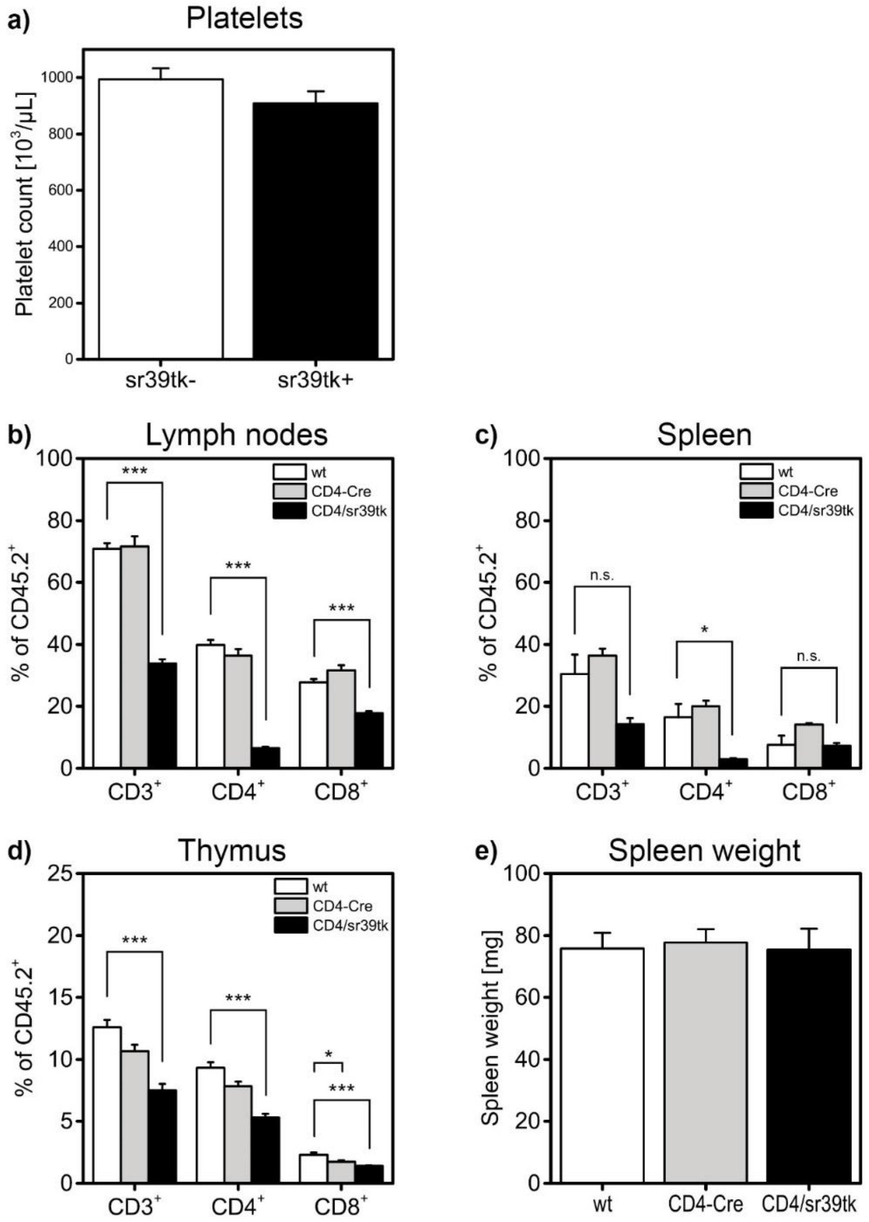

Supplementary Figure 4. Platelet counts in Pf4/sr39tk mice and T cell numbers in CD4/sr39tk mice. (**a**) Platelet counts in peripheral blood of 5 Pf4/sr39tk mice (sr39tk+; genotype: Cre[tg/+],R26[sr39tk/+]) and 5 Cre-negative control animals (sr39tk-; genotype: Cre[+/+],R26[sr39tk/+]). Data are shown as mean ± s.e.m of individual animals. (**b-d**) Flow cytometric analysis of T lymphocyte populations in (**b**) lymph nodes, (**c**) spleen, and (**d**) thymus of 4 wild type mice (wt; genotype: Cre[+/+],R26[+/+]), 4 CD4-Cre mice (genotype: Cre[tg/+],R26[+/+]), and 4 CD4/sr39tk mice (genotype: Cre[tg/+],R26[sr39tk/+]); '+' denotes the wild type allele. Cells were co-stained with antibodies against the leucocyte marker CD45.2 and the T cell markers CD3, CD4, and CD8 coupled to BD Horizon V500, allophycocyanin, BD Horizon V450, or fluorescein-isothiocyanate, respectively. Samples were analyzed with a flow cytometer using unstained cells as controls. One-way ANOVA was used to compare genotypes (* and *** indicate $p<0.05$ and $p<0.001$, respectively; n.s., not significant). (**e**) Comparison of spleen wet weights of the mice used for flow cytometry.

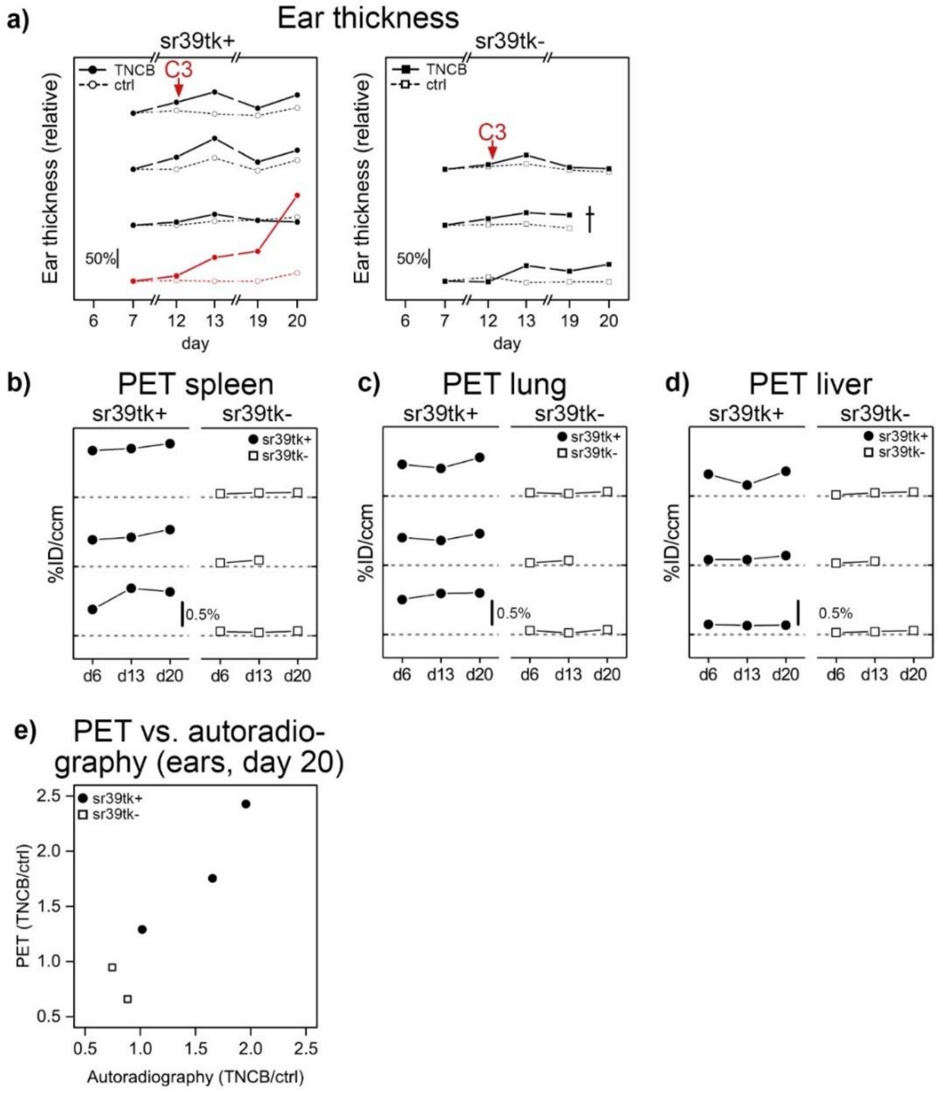

Supplementary Figure 5. Extended analysis of CD4/sr39tk mice subjected to DTHR. (**a**) Change in thickness of TNCB-challenged left ears (TNCB, solid lines) and non-challenged right ears (ctrl, dotted lines) assessed with a digital micrometer. Time courses of individual CD4/sr39tk mice (sr39tk+, left) and sr39tk- control animals (right) are shown; values were normalized to ear thickness before the first TNCB challenge on day 7. 'C3' indicates the third challenge with TNCB. One sr39tk+ animal (red) showed an abnormal increase in thickness of the left ear, most likely caused by bacterial superinfection of the TNCB-challenged left ear. This animal was excluded from the study. One sr39tk- animal died on day 20 under anesthesia (†); PET and *ex vivo* data for day 20 were not collected, but PET data obtained on day 6 and day 13 from this animal were included in the analysis. (**b-d**) Time course of [^{18}F]FHBG uptake in (**b**) spleen, (**c**) lung, and (**d**) liver of individual sr39tk+ (left) and sr39tk- (right) mice measured *in vivo* via PET imaging; values were normalized to injected dose (ID) and ROI volume (in ccm); grey dashed lines represent 0 %ID/ccm for each animal. (**e**) Correlation of [^{18}F]FHBG uptake measured *in vivo* by PET imaging and *ex vivo* by autoradiography. Data points represent the ratio of [^{18}F]FHBG uptake into the TNCB-challenged ear (TNCB) over the untreated ear (ctrl) of individual mice on day 20 of the study.

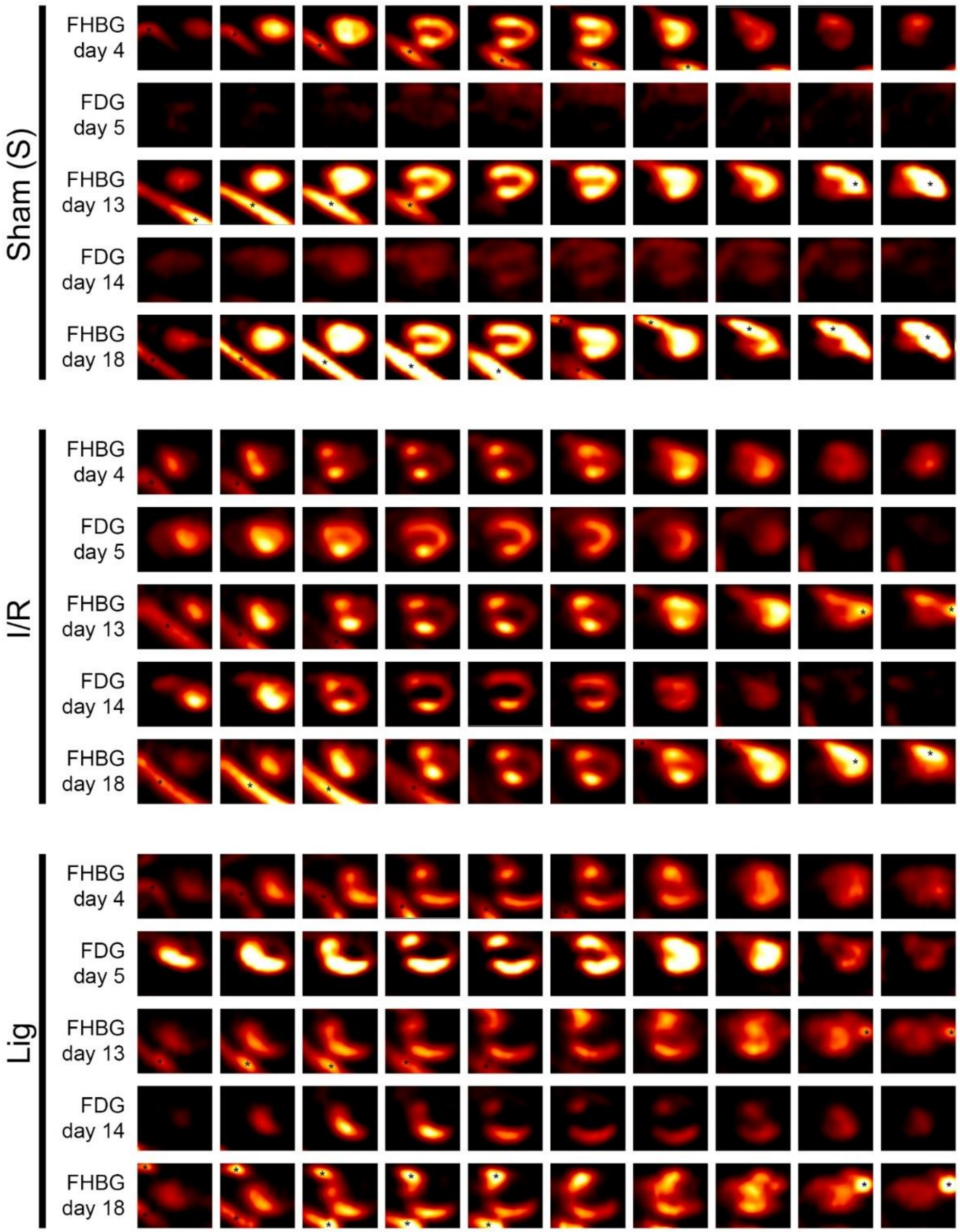

Supplementary Figure 6. Extended PET data for myocardial uptake of [^{18}F]FHBG and [^{18}F]FDG in Myh6i/sr39tk mice with myocardial infarction. Representative vertical long axis PET images of the left ventricle from medial (left) to lateral (right) of individual mice over the time course of the study (the same animals as in Fig. 5b are shown). Nonspecific signals in [^{18}F]FHBG images were derived from bone (*). Images were normalized to injected dose of the respective tracer. Myocardial infarction was induced by ischemia-reperfusion injury (I/R) or permanent LAD ligation (Lig), or mice underwent sham surgery (Sham).

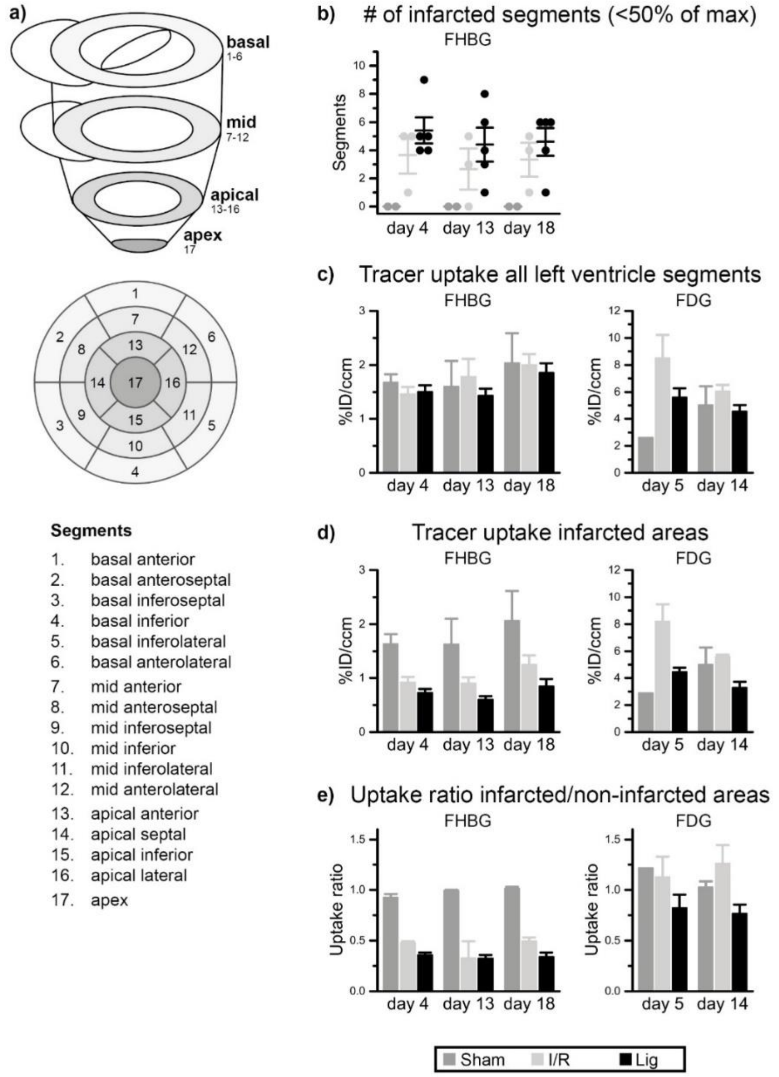

Supplementary Figure 7. Regional differences of tracer uptake into the heart of Myh6i/sr39tk mice with myocardial infarction. (**a**) Segmentation model[1] used to analyze regional differences in tracer uptake measured by PET imaging of the left ventricle. (**b-e**) Quantitative analysis of regional tracer uptake into the left ventricle of Myh6i/sr39tk mice at various times after MI. MI was induced by ischemia-reperfusion injury (I/R group, 3 mice) or permanent LAD ligation (Lig group, 5 mice), or mice underwent sham surgery (Sham group, 2 mice). (**b**) Estimation of infarct size given as number of segments with [^{18}F]FHBG uptake <50% of maximal tracer uptake into the left ventricle. Segments 7, 12, 13, 16, and 17 were most frequently infarcted. Data points represent individual animals, and horizontal bars represent mean ± s.e.m of all animals in the respective group. (**c**) Tracer uptake ([^{18}F]FHBG left, [^{18}F]FDG right) into all 17 segments of the left ventricle. (**d**) Tracer uptake ([^{18}F]FHBG left, [^{18}F]FDG right) into the infarcted area (defined as regions with <50% of maximal [^{18}F]FHBG uptake in the same animal). (**e**) Uptake ratio between infarcted and non-infarcted area ([^{18}F]FHBG left, [^{18}F]FDG right). The non-infarcted area was comprised of segments 2, 3, 8, and 9 in all animals and experiments. In the sham group, segments 7, 12, 13, 16, and 17 were formally defined as ‘infarcted’ area. Tracer uptake was normalized to injected dose (ID) and ROI volume (in ccm). Data are given as mean ± s.e.m of individual animals in the respective group.

Supplementary Table 1. Study parameters.

Experiment	Animals		Injected tracer (min-max)	Imaging	
**1. Cre lines**					
Pf4-Cre[2]	Cre promoter:<br>Age/Sex:<br>Genotype:<br>Treatment:	**Platelet factor 4 (CXCL4) gene**<br>10-14 weeks old; 8 male/2 female<br>4 Pf4/sr39tk, 6 sr39tk- controls<br>none	[^{18}F]FHBG<br>(11.5-13.6 MBq)	Anesthesia:<br>Uptake:<br>PET:<br>MRI: (optional)	Isoflurane<br>180 min conscious<br>20 min static<br>10 min T_2w
CD4-Cre[3]	Cre promoter:<br>Age/Sex:<br>Genotype:<br>Treatment:	**Cluster of differentiation 4 gene**<br>9-42 weeks old; 15 male/2 female<br>7 CD4/sr39tk, 10 sr39tk- controls<br>none (9) or TNCB-treatment (8)	[^{18}F]FHBG<br>(11.4-12.9 MBq)	Anesthesia:<br>Uptake:<br>PET:<br>MRI: (optional)	Isoflurane<br>180 min conscious<br>20 min static<br>10 min T_2w
Myh6-Cre[4]	Cre promoter:<br>Age/Sex:<br>Genotype:<br>Treatment:	**Myosin heavy chain 6 gene**<br>9-21 weeks old; 4 male/6 female<br>4 Myh6/sr39tk, 6 sr39tk- controls<br>none	[^{18}F]FHBG<br>(12.0-13.4 MBq)	Anesthesia:<br>Uptake:<br>PET:<br>MRI: (optional)	Isoflurane<br>180 min conscious<br>20 min static<br>10 min T_2w
**2. Inflammation** (CD4-Cre[3])	Age/Sex:<br>Genotype:<br>Treatment:	29-31 weeks old; 0 male/7 female<br>4 CD4/sr39tk, 3 sr39tk- controls<br>TNCB-treatment	[^{18}F]FHBG<br>(8.6-13.2 MBq)	Anesthesia:<br>Uptake:<br>PET:<br>MRI:	Isoflurane<br>180 min conscious<br>20 min static<br>10 min T_2w
**3. Myocardial infarction** (Myh6-CreERT2[5])	Age/Sex:<br>Genotype:<br>Treatment:	20-22 weeks old; 3 male/7 female<br>5 Myh6i/sr39tk, 5 Myh6i/sr39tk+lacZ<br>5 perm. Ligation (Lig), 3 I/R, 2 Sham	[^{18}F]FHBG<br>(10.7-11.2 MBq)	Anesthesia:<br>Uptake:<br>PET:	Isoflurane<br>180 min conscious<br>20 min static
			[^{18}F]FDG<br>(7.4-9.3 MBq)	Anesthesia:<br>Uptake:<br>PET:	Ketamine/Xylaxine<br>60 min unconscious<br>10 min static

SUPPLEMENTARY REFERENCES

1. Cerqueira, M.D., *et al.* Standardized myocardial segmentation and nomenclature for tomographic imaging of the heart. A statement for healthcare professionals from the Cardiac Imaging Committee of the Council on Clinical Cardiology of the American Heart Association. *Circulation* **105**, 539-542 (2002).

2. Tiedt, R., Schomber, T., Hao-Shen, H. & Skoda, R.C. Pf4-Cre transgenic mice allow the generation of lineage-restricted gene knockouts for studying megakaryocyte and platelet function in vivo. *Blood* **109**, 1503-1506 (2007).

3. Lee, P.P., *et al.* A critical role for Dnmt1 and DNA methylation in T cell development, function, and survival. *Immunity* **15**, 763-774 (2001).

4. Agah, R., *et al.* Gene recombination in postmitotic cells. Targeted expression of Cre recombinase provokes cardiac-restricted, site-specific rearrangement in adult ventricular muscle in vivo. *J Clin Invest* **100**, 169-179 (1997).

5. Takefuji, M., *et al.* G(13)-mediated signaling pathway is required for pressure overload-induced cardiac remodeling and heart failure. *Circulation* **126**, 1972-1982 (2012).
